# Educational Inequalities in Self-Rated Health in Europe and South Korea

**DOI:** 10.3390/ijerph17124504

**Published:** 2020-06-23

**Authors:** Minhye Kim, Young-Ho Khang, Hee-Yeon Kang, Hwa-Kyung Lim

**Affiliations:** 1 Institute of Health Policy and Management, Seoul National University Medical Research Center, 103 Daehak-ro (Yeongeon-dong) Jongno-gu, Seoul 03080, Korea; kmh8182@snu.ac.kr (M.K.); hklim@snu.ac.kr (H.-K.L.); 2 Ewha Institute for Age Integration Research, Ewha Womans University, SK Telecom building 504-1 ho, Ewhayeodae-gil 52, Seodaemun-gu, Seoul 03760, Korea; 3 Inequality and Social Policy Institute, Gacheon University, 1342 Seongnamdaero, Sujeong-gu, Seongnam-si, Gyeonggi-do 13120, Korea; 4 Department of Health Policy and Management, College of Medicine, Seoul National University, 103 Daehak-ro (Yeongeon-dong) Jongno-gu, Seoul 03080, Korea; hyeoni@snu.ac.kr

**Keywords:** cross-cultural comparison, health status disparities, European Union, Republic of Korea

## Abstract

While numerous comparative works on the magnitude of health inequalities in Europe have been conducted, there is a paucity of research that encompasses non-European nations such as Asian countries. This study was conducted to compare Europe and Korea in terms of educational health inequalities, with poor self-rated health (SRH) as the outcome variable. The European Union Statistics on Income and Living Conditions and the Korea National Health and Nutrition Examination Survey in 2017 were used (31 countries). Adult men and women aged 20+ years were included (207,245 men and 238,007 women). The age-standardized, sex-specific prevalence of poor SRH by educational level was computed. The slope index of inequality (SII) and relative index of inequality (RII) were calculated. The prevalence of poor SRH was higher in Korea than in other countries for both low/middle- and highly educated individuals. Among highly educated Koreans, the proportion of less healthy women was higher than that of less healthy men. Korea’s SII was the highest for men (15.7%) and the ninth-highest for women (10.4%). In contrast, Korea’s RII was the third-lowest for men (3.27), and the lowest among women (1.98). This high-SII–low-RII mix seems to have been generated by the high level of baseline poor SRH.

## 1. Introduction

Health inequalities have been observed in most of the world [1,2,3]. In South Korea (hereafter, Korea), health inequalities according to socioeconomic position (SEP) are reported to have emerged, and have increased in magnitude since the late 1980s [4]. Although all-cause mortality has decreased for the last 40 years, the disadvantage of those with low levels of education remains high [5]. Studies have shown that inequalities in other health outcomes, such as suicide and nutritional intake, also persist in Korea [6,7,8].

Despite the consistent findings on the association between SEP and health, there has been a crucial question with regard to the causality of the association: do differences in SEP cause differences in health? [9,10,11]. Compared with the associations of health with occupation or income, the association between education and health has been considered as more clearly suggesting causal associations free from health-related selection, since most of the health problems occur after the age at which people complete their education and thus the reverse causality (i.e., ill-health causes ill SEP) is unlikely to be involved [12,13]. Quasi-experimental studies showed that education reduces the risk of mortality and unhealthy behaviors [9,14,15]. Mendelian randomization studies, a technique casually examining the causal effect of modifiable factors on health and social outcomes using genes from the mother and father allocated at random to the child, also indicated the causal effect of more years of education on health behaviors, major diseases, and mortality [16,17,18].

International comparative studies on the magnitude of socioeconomic health inequalities may have very important implications. Based on the investigations on mortality inequalities by education and occupation among European countries, Mackenbach and colleagues concluded that countries whose social, economic, and healthcare policies have been more influenced by egalitarian principles were not successful in reducing relative mortality inequalities than other Southern European countries [1,19]. Studies on socioeconomic inequalities in mortality especially in European countries showed rather clear geographical patterns [20]. Inequalities in mortality have been reported to be clearly largest in Central-Eastern and Eastern Europe both in men and women while Southern European countries such as Italy and Spain have the smallest inequalities in mortality especially in women [20].

While numerous comparative works on health inequalities in European countries have been conducted [1,2,3,21,22], there is a paucity of research that encompasses non-European nations. International comparative studies on socioeconomic health inequalities including Asian welfare states such as Korea could provide important insight into the relationship between welfare arrangements and the magnitude of health inequalities. Korea and other Asian welfare states (e.g., Japan, Singapore, Taiwan) have been described as a “Confucian” welfare state regime characterized by a strong reliance on family and voluntary sector in providing safety nets and low levels of government expenditure on social welfare [23,24]. Very few comparative studies have explored those issues; however, the extant studies indicated that Korea’s health inequalities exhibit dissimilar features to those of European or other Asian societies: on the one hand, health inequalities in Korea were analyzed as being larger than those of other East Asian societies [25], while on the other hand, they were reported to be smaller than Western societies such as Europe and New Zealand [26,27]. More specifically, a study on inequalities in self-rated health (SRH) according to various SEP indicators in China, Japan, South Korea, and Taiwan revealed the greatest inequalities in SRH among Korean men (aged 20–69) in comparison to the other three East Asian societies [25]. In contrast, another study comparing occupational inequalities in mortality among men (aged 35–65) in Europe, Japan, and Korea argued that whereas European countries witness a clear occupational gradient in mortality, Korea sees a reversed pattern, with upper nonmanual workers having the greatest mortality and manual workers having the least mortality [26]. A study on New Zealand, England, Japan, and Korea also contended that inequalities in age-standardized cancer and cardiovascular disease mortality are relatively small in Korea [27]. A comparison among Organization for Economic Co-operation and Development (OECD) countries revealed that the difference of good SRH between the highest 20% and the lowest 20% income groups was one of the smallest in Korea [28].

As health inequalities in Korea as compared to European countries remain under-investigated, this study was conducted to compare the magnitude of educational inequalities in poor self-rated health in Europe and Korea. This study will aid in understanding the international patterns of the educational health inequalities in poor self-rated health.

## 2. Materials and Methods

### 2.1. Ethical Approval

This article does not present information from any studies with human participants performed by any of the authors. This study was approved by the Seoul National University Hospital Institutional Review Board (IRB No. E-1901-021-999).

### 2.2. Data

The European Union Statistics on Income and Living Conditions (EU-SILC) and the Korea National Health and Nutrition Examination Survey (KNHANES) in 2017 were used. A total of 31 countries were examined. These two datasets are known for their high quality and reliability: nationally representative, used as core data sources for producing OECD health statistics across European countries and Korea [28], and are frequently relied upon for comparisons of health inequalities in Europe [22,29]. This study extended the OECD reports in that education, more reliable SEP variable in terms of causality, was used and the most recent datasets were utilized. Adult men and women aged 20+ years with valid values for SRH, education, and survey weight were included (207,245 men and 238,007 women; Table 1).

### 2.3. Variables

SRH was rated with a question, “How is your health in general?” in Europe, and “In general, what do you think that your health is?” in Korea. In both datasets, there were five responses: “very good”, “good”, “fair”, “poor”, and “very poor.” To make the meaning clearer, responses were dichotomized as poor SRH (“poor” + “very poor”) and fair/good SRH (“fair” + “good” + “very good”), following prior studies which compared self-rated health across countries [3,4,21,22,25,28].

SEP was measured by the highest educational level attained. Among the many variables that can be used to quantify SEP, education is known to be the most reliable in terms of minimizing the likelihood of reverse causality and having high comparability across nations [22]. For analysis in this study, education was dichotomized as low/middle and high education [32]. Low/middle education referred to the completion of elementary, secondary, and postsecondary nontertiary levels of schooling. Elementary level referred to primary education such as primary school or elementary school. Secondary level referred to secondary school, junior secondary school, middle school, junior high school, senior secondary school, or (senior) high school. Postsecondary nontertiary levels included technician diploma or primary professional education. High education referred to the completion of tertiary-level education, which covered academic education equal or more than a Bachelor’s degree, or advanced vocational or professional education.

Age was calculated by subtracting the year of birth plus a year from 2017 based on the guideline by Eurostat [33]. As in the major literature with similar research design, age was treated as the main confounder [34,35,36].

### 2.4. Statistical Analysis

**Figure 1:**
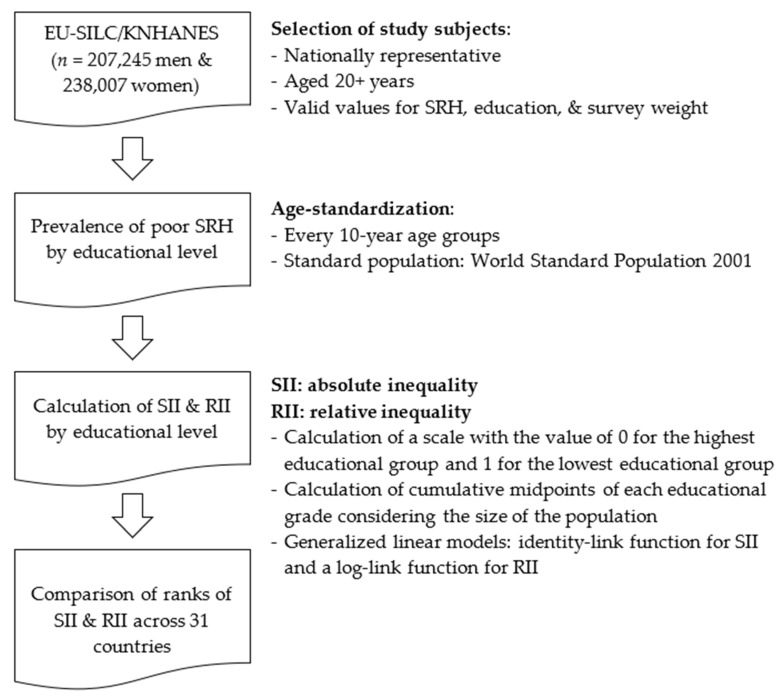
A graphical summary of the analysis.

The first step of the statistical analysis involved age-standardization of the prevalence of poor SRH (“poor” + “very poor”) by educational level (elementary/secondary and tertiary) for men and women in the 31 countries (Figure 1). Age-standardization was performed with 10-year age groups, using the World Standard Population 2001 as the standard population [37].

The second step was the calculation of the slope index of inequality (SII, absolute inequality) and the relative index of inequality (RII, relative inequality). These two measures are succinct and effective summary indicators of health inequality cross-nationally, as well as within society [34,35,36]. To obtain SII and RII, we first created a scale with the value of 0 for the highest educational group, and 1 for the lowest educational group for each country, sex, and age group (every 20 years). Considering the percentages of the population belonging to each educational category, cumulative midpoints of each educational grade were calculated. For instance, if 20–39-year-old highly educated Korean men comprised 30% of this age group, those who were in this group would have a value of 0.15 (0.30 × 0.5), while low/middle-educated men in this age group would have a value of 0.65 (0.30 + 0.70 × 0.5). Then, generalized linear models were used, with an identity-link function for SII and a log-link function for RII [34,35,36]. The models for SII and RII included the ridit score of education, controlling for age and age-squared:

g(*Y*) = *β*_1_*ridit* + *β*_2_*age* + *β*_3_*age*^2^ + *constant* + *error* (1)

where g(*Y*) is a link function, *Y* is 1 for poor health and 0 for fair/good health, and the error term follows a binomial distribution. The SII and RII can be calculated as *β*_1_ for the model with the identity-link function and *exp*(*β*_1_) for the model with the log-link function. The SII is interpreted as the prevalence difference (%) between the hypothetically lowest and highest educational groups. In a similar vein, the RII refers to the prevalence ratio (unitless) of poor SRH between the two extreme educational groups. Confidence intervals (CIs) at 95% were estimated. More details about the calculation of the SII and RII can be found elsewhere [38,39].

We used PROC SURVEYREG for age-standardized prevalence and PROC GENMOD for SII/RII in SAS version 9.4 for Windows (SAS, Cary, NC, USA). The comprehensive SAS syntax can be found in prior papers [40,41]. As this study utilized social surveys with individuals as the unit of analysis and cross-sectional comparison as the main focus, personal cross-sectional weights were taken into consideration by including syntax adjusting for this weight (PB040 in EU-SILC and wt_itvex in KNHANES) in all statistical analyses.

## 3. Results

**Figure 2:**
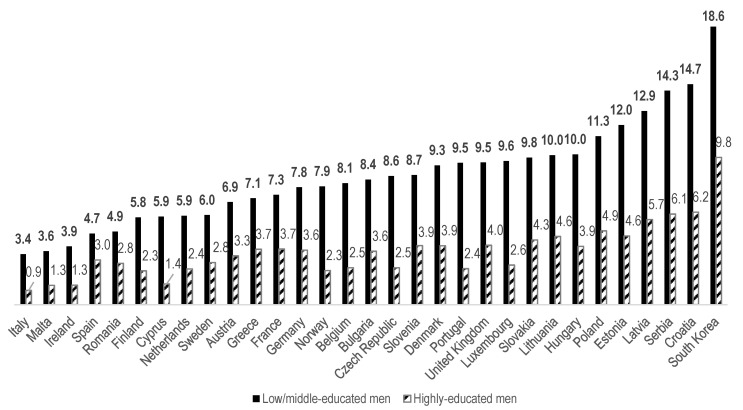
Age-standardized poor (poor + very poor) self-rated health (SRH, %) for low/middle- and highly educated adult men (aged 20+) in 2017, in ascending order by prevalence among the less-educated.

**Figure 3:**
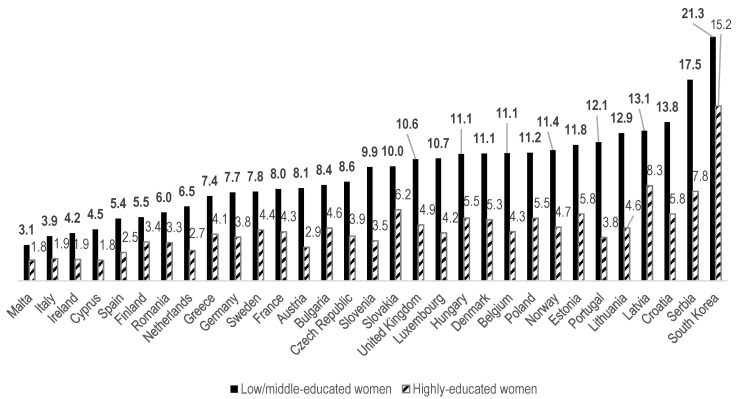
Age-standardized poor (poor + very poor) self-rated health (SRH, %) for low/middle- and high-educated adult women (aged 20+) in 2017, in ascending order by prevalence among the less-educated.

Figure 2 and Figure 3 present the age-standardized prevalence of poor SRH among 31 countries. Korea had a relatively high age-standardized prevalence of poor SRH. All the prevalence rates of poor SRH among low/middle- and highly educated men and women were higher in Korea than in any other country. Among low/middle-educated men, the value was 18.6% in Korea, followed by 14.7% in Croatia and 14.3% in Serbia (Figure 2). Similarly, for highly educated men, the greatest prevalence was found in Korea (9.8%), followed by Croatia (6.2%) and Serbia (6.1%, Figure 2).

The age-standardized prevalence of poor SRH among low/middle-educated women was 21.3% in Korea, followed by 17.5% in Serbia and 13.8% in Croatia (Figure 3). For highly educated women, the figure was 15.2% in Korea, followed by Latvia (8.3%) and Serbia (7.8%, Figure 3). It seems that highly educated women’s disproportionately pervasive self-rated ill-health in Korea was fairly distinctive. The prevalence of poor SRH among highly educated Korean women was 6.9% point (p) higher than the second-highest prevalence (Latvia). In contrast, the prevalence of poor SRH among highly educated Korean men showed a relatively moderate gap, as it was only 3.5%p higher than that of the next country (Croatia). Furthermore, the difference between men and women within Korea was also noteworthy, as the prevalence of poor SRH among highly educated women was much higher (15.2%) than among their male counterparts (9.8%), despite equally high levels of education in both groups.

**Figure 4:**
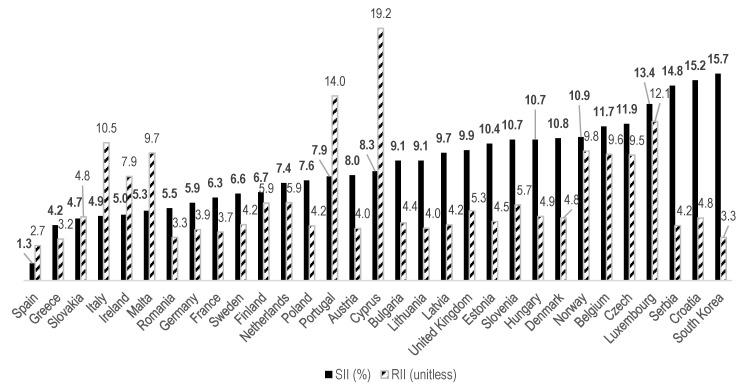
SIIs and RIIs among men, in ascending order by SII.

**Figure 5:**
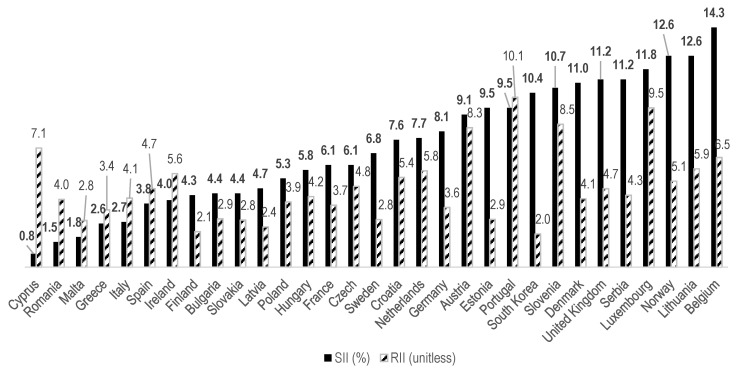
SIIs and RIIs among women, in ascending order by SII.

The absolute educational inequality in SRH in men, measured by the SII, was highest in Korea (15.7%; 95% CI: 9.9–21.5%), followed immediately by Croatia (15.2%; 95% CI: 11.3–19.0%) and Serbia (14.8%; 95% CI: 8.7–20.8%, Table 2, Figure 4). In contrast, among women, Korea showed the ninth-highest SII among the 31 countries, with a value of 10.4% (95% CI: 4.4–16.4%). The closest value to Korea was observed for Slovenia (10.7%; 95% CI: 7.7–13.8%, Table 3, Figure 5).

The relative educational inequalities in SRH, expressed through the RII, demonstrated a rather dissimilar picture to the results obtained for absolute inequalities. For men, Korea was the third most equal country (3.27, 95% CI: 2.15–4.99), following Spain (2.66, CI: 1.81–3.91) and Greece (3.15, CI: 2.44–4.06, Table 2, Figure 4). The RII for women was even lower (1.98; 95% CI: 1.38–2.85), making Korea the most equal among the 31 societies (Table 3, Figure 5). The second-lowest was Finland (2.12; 95% CI: 1.22–3.71). This combination of relatively high absolute and comparatively low relative inequalities sheds light on the characteristics of the current state of health equity in Korea as compared to European countries.

## 4. Discussion

This study aimed to situate the degree of educational inequalities in health in Korea relative to Europe by comparing the age-standardized prevalence of poor SRH, as well as absolute and relative indices of inequality (SII and RII), among adult men and women. Two nationally representative datasets (EU-SILC and KNHANES), which are used to generate major OECD statistics, were utilized.

The results of this study revealed that educational inequalities in SRH existed in all 31 countries, including Korea, and in both men and women. These results are in line with prior investigations in that lower SEP corresponds to poorer SRH in most of the world [1,2,3,4,21,22,25]. The results that all of the 31 countries included in this analysis showed educational health inequality indicates the importance of global efforts to reduce health inequalities. A comparison of the RIIs of educational inequalities in SRH reported in a prior paper [1] with those computed in this study for the same European countries examined yielded a Spearman correlation coefficient of 0.610 (*p* = 0.006) for men and 0.500 (*p* = 0.029) for women. Differences in the survey period (2015 versus circa 2000), the data (EU-SILC versus various health and social surveys) and outcome variables (poor SRH versus a weighted SRH variable) might have resulted in the less than excellent between-study correlations for educational inequalities in SRH.

Prior comparative studies on educational mortality inequalities among European countries presented geographic patterns [1]. The magnitude of educational inequalities in mortality was generally smaller than the average for Europe in all Southern European populations and larger than average in most countries in the eastern and Baltic regions [1]. A recent study on mortality inequalities in Europe also showed similar patterns [20]. However, the international pattern in Europe on inequalities in SRH from this study tended to be different from the pattern found for mortality. For example, the magnitude of relative inequalities in SRH among men was smaller in Spain than in any other country, but adult men in Italy recorded a very large educational inequality in SRH. Study results indicated that, among adult men, some eastern European countries such as Lithuania, Poland, and Romania recorded fairly smaller relative inequalities in SRH by education than many other European countries.

In terms of the age-standardized prevalence of poor SRH, Korea ranked highest among all educational groups for both men and women out of the 31 countries. Korea is well-known to have generally high levels of poor SRH, one of the highest among OECD nations [42]. In addition, the prevalence of less than good SRH has been steadily increasing for the last three decades in Korea [4]. For men, the prevalence of less than good SRH for those with a college education or higher was 35.9% in 1989, and rose to 44.5% in 1999. The percentages for less-educated men and women and for highly educated women have changed similarly (for example, from 45.5% in 1989 to 60.3% in 1999 for men with an elementary school education or less). Accordingly, men’s RII for less than good SRH by education was as small as 1.67; and women’s RII was even less than one (0.96) in 1989 [4]. This indicates that educational health inequalities were small among men and did not exist among women in 1989, although this pattern had changed dramatically toward greater inequalities among both men (RII = 2.26) and women (RII = 1.94) by 1999. Although those figures cannot be directly compared to the findings of this study due to differences in the age spans of respondents and a dissimilar categorization of responses of SRH, the results of this study (3.27 for men and 1.98 for women in terms of RII) suggest even stronger inequalities. Therefore, the results of this study seem to reflect the general pattern of mounting prevalence of poor SRH in Korea.

The point made above is closely related to the next point: Korea’s absolute differences (SIIs) in poor SRH were comparatively high, while the relative inequalities (RIIs) of poor SRH were on the lower side for both men and women. Among the countries studied, the SII in Korea was the highest among men and the ninth-highest among women. In contrast, the RII was the third-lowest among men and the lowest among women. This high-SII–low-RII mix seems to have been generated by the high baseline prevalence of poor SRH, as discussed earlier. As the prevalence of poor SRH in Korea is high, regardless of sex and SEP, it is likely that the SII, as a kind of weighted difference in rates, is largely due to the large absolute numbers, whereas the RII, as a sort of weighted ratio of rates, is small due to the large denominators. Other countries with high SII also exhibit a low RII [22].

A similar comparison based on income level reported that the difference in the percentage of respondents with self-rated good health among the fifth quintile (highest 20% of income) and the first quintile (lowest 20%) in Korea was 10.6% in 2013, which was one of the lowest differences among OECD countries [28]. As the percentage of individuals with good SRH is the inverse of that of individuals with poor SRH, the prevalence was generally low for both of the fifth and first quintiles, which is why the percentage difference was one of the smallest in Korea.

As for the reason why health inequalities in SRH have emerged and become amplified in Korea, studies have suggested that the expansion of the national health insurance system has enabled less-educated individuals to detect illnesses and diseases more easily [4]. Korea’s universal health coverage started in 1989; correspondingly, the prevalence of poor SRH particularly surged from 1989 to 1992. Inequalities further expanded during the economic crisis (1995–1999). The comparatively very high poor SRH in this study suggests that this trend has not necessarily diminished.

A different composition of prevalent diseases may have resulted in the differences between Korea with Europe. According to the literature, cardiovascular disease is the leading contributor to inequalities in mortality in Europe, whereas cancer is the most important contributor in Korea [26]. It is worth highlighting that socioeconomic inequalities in cancer tend to be smaller than those of cardiovascular diseases, thereby reducing the RII in countries such as Korea in comparison to other societies such as Europe.

Self-rated health consists of two dimensions: objective health problems and the perception and reporting styles on those health problems. Studies using “anchoring vignettes” provided evidence on the differences between objective measurements and self-reports of health problems [43,44,45,46]. In addition, inequalities in SRH might be affected by reporting styles. Several prior studies from Europe, Indonesia, and South Africa showed that highly educated or rich respondents tended to respond more negatively than lower educated or poor respondents, and, as a result, inequalities in SRH are likely to underestimate true differences in health status [44,45,46]. However, no study has been conducted in Korea to explore the difference in the magnitude of inequalities between objective health problems and self-reports of those problems. Further research will be necessary regarding whether the differences in educational health inequalities found between Europeans and Koreans in this study originated from objective health problems or subjective perceptions towards them.

This study provides a more comprehensive picture of educational health inequalities in Korea by juxtaposing Korean and European data. However, although maximum efforts were exerted to synchronize the two datasets, some comparability issues remain. For instance, the 31 countries in this study contain different numbers of respondents and slightly dissimilar age spans. The survey method also has limitations. In particular, as surveys do not include the entire population and depend on self-reporting, they can be less accurate than administrative data. Cross-cultural comparisons based on more robust data sources such as census, death certificates, or big data are encouraged. In addition, in further studies, it will be more informative to include more varied types of variables (e.g., smoking status as a mediator for the association between education and poor SRH) other than age. Considering that Korea’s health inequalities have been increasing [21,47], cross-sectional investigations should be extended into longitudinal analyses by tracking trends using multiple waves of EU-SILC and KNHANES, which will delineate temporal as well as geographical configurations of health inequalities. This study used education as the focal SEP, which demonstrated clearer causality in terms of the association with health inequality than other variables such as occupation and income. However, this does not warrant that the causal effect of education on health inequality is proved.

In conclusion, educational inequalities in poor SRH were evident in all 31 countries, including Korea. The absolute magnitude of educational inequalities in poor SRH, measured by the SII, was highest for Korean men and the ninth-highest for Korean women. However, the relative magnitude of educational inequalities was relatively small for Korean men and women. Further international comparative studies across high-income countries, including Korea, are required to provide additional information on the magnitude and ranking of health inequalities and associated determinants.

## Figures and Tables

**Table 1:** Characteristics of study subjects in 31 countries.

Country	Response Rate ^1^ (%)	Men				Women			
		Number of Respondents	Mean Age	Low/ Middle- Educated (%)	Highly Educated (%)	Number of Respondents	Mean Age	Low/ Middle- Educated (%)	Highly Educated (%)
Austria	72.2	4825	50.9	63.2	36.8	5422	51.6	71.5	28.5
Belgium	59.2	4883	49.9	62.8	37.2	5286	50.5	61.1	38.9
Bulgaria	85.0	6879	52.4	82.8	17.2	7909	55.9	76.6	23.4
Croatia	63.7	7761	52.0	84.0	16.0	8637	54.4	83.1	16.9
Cyprus	84.8	4219	50.0	70.7	29.3	4830	50.6	69.1	30.9
Czech Republic	75.4	4479	54.5	80.8	19.2	6847	55.5	81.9	18.1
Denmark	63.9	2701	55.4	66.0	34.0	2914	55.8	59.4	40.6
Estonia	78.2	3568	52.2	72.8	27.2	5179	54.3	60.3	39.7
Finland	76.2	4527	51.3	64.2	35.8	4447	52.1	54.6	45.4
France	74.6	8840	51.6	71.5	28.5	9870	52.7	69.6	30.5
Germany	77.3	10,413	52.9	58.3	41.8	11,642	52.7	72.1	27.9
Greece	87.7	21,451	53.4	77.3	22.7	23,025	54.7	78.9	21.1
Hungary	82.4	6600	51.5	83.8	16.2	8319	55.2	82.9	17.1
Ireland	57.0	4263	52.0	59.8	40.3	4640	52.1	59.1	40.9
Italy	74.1	18,598	52.4	84.6	15.5	20,653	54.4	84.5	15.5
Latvia	74.4	4319	50.7	79.4	20.7	6090	56.1	68.3	31.7
Lithuania	71.9	2509	54.0	74.2	25.8	4373	56.6	69.3	30.7
Luxembourg	48.5	3916	46.8	71.0	29.0	4101	46.9	71.3	28.7
Malta	84.1	4065	48.5	83.2	16.8	4233	50.3	82.9	17.1
Netherlands	51.9	5674	53.5	61.7	38.3	7104	54.1	66.3	33.7
Norway	53.5	2950	49.6	59.5	40.5	2834	50.4	53.2	46.8
Poland	N.A.	10,939	50.7	82.1	17.9	13,552	53.0	77.0	23.0
Portugal	86.0	11,204	51.7	87.5	12.5	13,125	53.4	81.7	18.3
Romania	92.6	7106	51.8	87.9	12.1	7829	54.1	88.0	12.0
Serbia	N.A.	6494	49.4	84.3	15.7	6966	51.7	83.8	16.3
Slovakia	84.1	6048	48.1	81.5	18.5	6948	51.1	78.5	21.5
Slovenia	68.1	3939	50.4	76.6	23.4	4441	52.7	70.9	29.1
South Korea	77.9	2543	51.5	57.9	42.2	3224	52.3	64.6	35.4
Spain	71.9	13,136	51.2	71.4	28.6	14,465	53.0	70.8	29.2
Sweden	50.8	2718	52.2	67.2	32.8	2790	52.8	55.3	44.7
United Kingdom	48.3	5678	55.8	59.6	40.4	6312	54.6	59.9	40.1

Note: ^1^—The ratio of achieved sample size to actual sample size based on Eurostat [30] and Korea Centers for Disease Control and Prevention [31]; N.A.—not available.

**Table 2:** Age-standardized poor (poor + very poor) self-rated health (%) for the low/middle- and highly educated, absolute inequality (slope index of inequality (SII), %), and relative inequality (relative index of inequality (RII), unitless) among adult men aged 20+ by country in 2017.

Country	Low/ Middle- Educated *	Highly Educated	SII (%)	(95% CI)	Rank of SII	RII	(95% CI)	Rank of RII
**Italy**	3.4	0.9	4.9%	(3.5–6.3%)	4	10.46	(5.35–20.47)	28
**Malta**	3.6	1.3	5.3%	(−1.7–12.3%)	6	9.68	(2.25–41.53)	26
**Ireland**	3.9	1.3	5.0%	(3.1–7.0%)	5	7.90	(3.36–18.58)	23
**Spain**	4.7	3.0	1.3%	(0.2–2.3%)	1	2.66	(1.81–3.91)	1
**Romania**	4.9	2.8	5.5%	(0.8–10.2%)	7	3.27	(1.46–7.29)	3
**Finland**	5.8	2.3	6.7%	(3.7–9.8%)	11	5.88	(3.05–11.32)	21
**Cyprus**	5.9	1.4	8.3%	(3.7–12.8%)	16	19.15	(7.11–51.56)	31
**Netherlands**	5.9	2.4	7.4%	(4.6–10.1%)	12	5.91	(3.35–10.44)	22
**Sweden**	6.0	2.8	6.6%	(3.5–9.7%)	10	4.24	(1.72–10.47)	12
**Austria**	6.9	3.3	8.0%	(4.7–11.4%)	15	3.96	(2.38–6.59)	7
**Greece**	7.1	3.7	4.2%	(3.4–5.0%)	2	3.15	(2.44–4.06)	2
**France**	7.3	3.7	6.3%	(4.6–8.0%)	9	3.68	(2.41–5.64)	5
**Germany**	7.8	3.6	5.9%	(4.2–7.7%)	8	3.87	(2.82–5.29)	6
**Norway**	7.9	2.3	10.9%	(6.2–15.5%)	25	9.83	(4.34–22.25)	27
**Belgium**	8.1	2.5	11.7%	(8.1%15.3%)	26	9.58	(5.45–16.85)	25
**Bulgaria**	8.4	3.6	9.1%	(5.6–12.7%)	17	4.38	(2.71–7.08)	13
**Czech Republic**	8.6	2.5	11.9%	(5.6–18.1%)	27	9.52	(4.67–19.38)	24
**Slovenia**	8.7	3.9	10.7%	(5.3–16.1%)	22	5.74	(3.19–10.31)	20
**Denmark**	9.3	3.9	10.8%	(7.0–14.6%)	24	4.75	(2.46–9.15)	15
**Portugal**	9.5	2.4	7.9%	(6.4–9.4%)	14	14.01	(7.86–24.97)	30
**United Kingdom**	9.5	4.0	9.9%	(7.1–12.6%)	20	5.28	(3.49–7.98)	19
**Luxembourg**	9.6	2.6	13.4%	(6.8–20.0%)	28	12.07	(6.05–24.07)	29
**Slovakia**	9.8	4.3	4.7%	(3.1–6.3%)	3	4.83	(2.77–8.44)	17
**Hungary**	10.0	3.9	10.7%	(7.5–13.9%)	22	4.87	(3.12–7.62)	18
**Lithuania**	10.0	4.6	9.1%	(2.4–15.8%)	17	3.97	(2.17–7.27)	8
**Poland**	11.3	4.9	7.6%	(6.0–9.2%)	13	4.16	(2.96–5.87)	9
**Estonia**	12.0	4.6	10.4%	(7.6–13.3%)	21	4.48	(2.90–6.93)	14
**Latvia**	12.9	5.7	9.7%	(6.3–13.2%)	19	4.23	(2.71–6.59)	11
**Serbia**	14.3	6.1	14.8%	(8.7–20.8%)	29	4.17	(2.93–5.92)	10
**Croatia**	14.7	6.2	15.2%	(11.3–19.0%)	30	4.75	(3.38–6.69)	15
**South Korea**	18.6	9.8	15.7%	(9.9–21.5%)	31	3.27	(2.15–4.99)	3

Note: *—Ascending order by prevalence among the less-educated.

**Table 3:** Age-standardized poor (poor + very poor) self-rated health (%) for the low/middle- and highly educated, absolute inequality (slope index of inequality (SII), %), and relative inequality (relative index of inequality (RII), unitless) among adult women aged 20+ by country in 2017.

Country	Low/ Middle- Educated *	Highly Educated	SII (%)	(95% CI)	Rank of SII	RII	(95% CI)	Rank of RII
**Malta**	3.1	1.8	1.8%	(−1.6–5.2%)	3	2.79	(0.73–10.63)	4
**Italy**	3.9	1.9	2.7%	(1.9–3.5%)	5	4.12	(2.47–6.87)	15
**Ireland**	4.2	1.9	4.0%	(1.9–6.1%)	7	5.58	(2.64–11.81)	23
**Cyprus**	4.5	1.8	0.8%	(−0.7–2.3%)	1	7.11	(2.63–19.21)	27
**Spain**	5.4	2.5	3.8%	(2.5–5.2%)	6	4.68	(3.06–7.16)	18
**Finland**	5.5	3.4	4.3%	(1.8–6.7%)	8	2.12	(1.22–3.71)	2
**Romania**	6	3.3	1.5%	(0.6–2.4%)	2	4.04	(1.78–9.17)	13
**Netherlands**	6.5	2.7	7.7%	(4.8–10.5%)	18	5.75	(3.37–9.80)	24
**Greece**	7.4	4.1	2.6%	(1.8–3.4%)	4	3.42	(2.54–4.60)	9
**Germany**	7.7	3.8	8.1%	(5.8–10.3%)	19	3.56	(2.41–5.25)	10
**Sweden**	7.8	4.4	6.8%	(3.0–10.6%)	16	2.84	(1.51–5.34)	6
**France**	8	4.3	6.1%	(4.1–8.1%)	14	3.7	(2.56–5.34)	11
**Austria**	8.1	2.9	9.1%	(6.8–11.4%)	20	8.32	(4.38–15.79)	28
**Bulgaria**	8.4	4.6	4.4%	(2.8–6.0%)	9	2.88	(2.04–4.08)	8
**Czech Republic**	8.6	3.9	6.1%	(3.9–8.3%)	14	4.82	(2.80–8.31)	20
**Slovenia**	9.9	3.5	10.7%	(7.7–13.8%)	24	8.53	(4.86–14.98)	29
**Slovakia**	10	6.2	4.4%	(2.8–6.1%)	9	2.81	(1.78–4.45)	5
**United Kingdom**	10.6	4.9	11.2%	(8.4–14.0%)	26	4.68	(3.23–6.78)	18
**Luxembourg**	10.7	4.2	11.8%	(8.3–15.4%)	28	9.5	(4.98–18.09)	30
**Belgium**	11.1	4.3	14.3%	(10.4–18.1%)	31	6.54	(4.22–10.14)	26
**Denmark**	11.1	5.3	11.0%	(7.0–15.0%)	25	4.08	(2.40–6.95)	14
**Hungary**	11.1	5.5	5.8%	(3.7–7.9%)	13	4.21	(2.93–6.07)	16
**Poland**	11.2	5.5	5.3%	(3.7–6.9%)	12	3.89	(2.90–5.22)	12
**Norway**	11.4	4.7	12.6%	(8.5–16.8%)	29	5.13	(2.80–9.39)	21
**Estonia**	11.8	5.8	9.5%	(6.6–12.4%)	21	2.85	(2.15–3.78)	7
**Portugal**	12.1	3.8	9.5%	(7.5–11.6%)	21	10.13	(6.93–14.81)	31
**Lithuania**	12.9	4.6	12.6%	(8.0–17.2%)	29	5.87	(3.90–8.83)	25
**Latvia**	13.1	8.3	4.7%	(1.8–7.5%)	11	2.4	(1.86–3.10)	3
**Croatia**	13.8	5.8	7.6%	(5.3–9.9%)	17	5.35	(3.70–7.74)	22
**Serbia**	17.5	7.8	11.2%	(8.0–14.3%)	26	4.29	(3.05–6.03)	17
**South Korea**	21.3	15.2	10.4%	(4.4–16.4%)	23	1.98	(1.38–2.85)	1

Note: *−Ascending order by prevalence among the less-educated.
